# Paramyxovirus replication induces the hexosamine biosynthetic pathway and mesenchymal transition via the IRE1α-XBP1s arm of the unfolded protein response

**DOI:** 10.1152/ajplung.00127.2021

**Published:** 2021-07-28

**Authors:** Dianhua Qiao, Melissa Skibba, Xiaofang Xu, Roberto P. Garofalo, Yingxin Zhao, Allan R. Brasier

**Affiliations:** ^1^Department of Internal Medicine, University of Wisconsin-Madison School of Medicine and Public Health, Madison, Wisconsin; ^2^Department of Pediatrics, University of Texas Medical Branch, Galveston, Texas; ^3^Department of Internal Medicine, University of Texas Medical Branch, Galveston, Texas; ^4^Institute for Clinical and Translational Research, University of Wisconsin-Madison, Madison, Wisconsin

**Keywords:** epithelial mesenchymal transition, hexosamine biosynthetic pathway, N-glycosylation, unfolded protein response, paramyxovirus

## Abstract

The paramyxoviridae, respiratory syncytial virus (RSV), and murine respirovirus are enveloped, negative-sense RNA viruses that are the etiological agents of vertebrate lower respiratory tract infections (LRTIs). We observed that RSV infection in human small airway epithelial cells induced accumulation of glycosylated proteins within the endoplasmic reticulum (ER), increased glutamine-fructose-6-phosphate transaminases (GFPT1/2) and accumulation of uridine diphosphate (UDP)-*N*-acetylglucosamine, indicating activation of the hexosamine biosynthetic pathway (HBP). RSV infection induces rapid formation of spliced X-box binding protein 1 (XBP1s) and processing of activating transcription factor 6 (ATF6). Using pathway selective inhibitors and shRNA silencing, we find that the inositol-requiring enzyme (IRE1α)-XBP1 arm of the unfolded protein response (UPR) is required not only for activation of the HBP, but also for expression of mesenchymal transition (EMT) through the Snail family transcriptional repressor 1 (SNAI1), extracellular matrix (ECM)-remodeling proteins fibronectin (FN1), and matrix metalloproteinase 9 (MMP9). Probing RSV-induced open chromatin domains by ChIP, we find XBP1 binds and recruits RNA polymerase II to the *IL6*, *SNAI1*, and *MMP9* promoters and the intragenic superenhancer of glutamine-fructose-6-phosphate transaminase 2 (GFPT2). The UPR is sustained through RSV by an autoregulatory loop where XBP1 enhances Pol II binding to its own promoter. Similarly, we investigated the effects of murine respirovirus infection on its natural host (mouse). Murine respirovirus induces mucosal growth factor response, EMT, and the indicators of ECM remodeling in an IRE1α-dependent manner, which persists after viral clearance. These data suggest that IRE1α-XBP1s arm of the UPR pathway is responsible for paramyxovirus-induced metabolic adaptation and mucosal remodeling via EMT and ECM secretion.

## INTRODUCTION

The paramyxoviridae are species-adapted, enveloped negative-sense RNA viruses responsible for respiratory tract infections in vertebrates. In humans, respiratory syncytial virus (RSV) is responsible for seasonal outbreaks of respiratory tract infections worldwide (1). RSV is the most common cause of pediatric hospitalization in children less than 5 yr of age (2) and responsible for one-third of lower respiratory tract infections (LRTIs) globally (3). In immunologically naïve children, RSV-induced lower respiratory tract infection (LRTI) results in epithelial giant cell formation and necrosis, producing mucous plugging, ventilation-perfusion mismatching, and acute hypoxic respiratory failure (4). In rodents, murine respirovirus is a highly permissive infection (5) that produces airway inflammation, giant cell formation, and necrosis of the respiratory epithelium (6), features shared with those in RSV LRTI.

Upon inoculation, both RSV and murine respirovirus replicate in ciliated airway epithelial cells in the upper nasopharynx and conducting airways (7, 8), spreading into the lower airways to produce persistent inflammation (9). Through an interconnected network of cell-surface and cytoplasmic pattern recognition receptors, RSV replication triggers a robust innate antiviral response consisting of secretion of cytokine (10, 11), interferon (12), and damage-associated patterns (13). These factors act in paracrine manner to initiate leukocytic inflammation (13–15), mucous production (16), and reduce viral replication (17).

Prospective observational studies of children with severe RSV infections show that LRTIs are associated with long-term decreased pulmonary function through largely unknown mechanisms (18, 19). The mechanisms of how viral exposures may be linked to structural remodeling are underexplored. Although RSV productively replicates in epithelial cells from the nasal, conductive, and lower airways (4), recent work has indicated that the qualitative patterns of inducible genomic and proteomic response of these cell types are distinct and linked to distinct manifestations of disease (20). In particular, small airway epithelial cells (SAECs) derived from secretoglobin (*Scg1a1*^+^)-expressing progenitors are uniquely primed to induce neutrophilic, T helper 2 (Th2)-polarizing, and mucogenic cytokines that mediate characteristic pathology of LRTIs (18, 21). Moreover, human small airway epithelial cells (hSAECs) are programmed to enter cell state changes upon growth factor stimulation (22). These intriguing data inform the hypothesis that injury-repair processes activated in small airway epithelial cells play a role in the extracellular matrix remodeling in response to viral LRTI.

In small airway epithelial cells, RSV replication induces profound cellular metabolic adaptations. Metabolic profiling studies have shown that RSV replication upregulates glucose influx, aerobic glycolysis, and increased lactic acid generation (23, 24). This shift of cellular metabolism towards aerobic glycolysis is characteristic of that seen in malignancy and known as the Warburg effect (25). In addition, RSV infection induces accumulation of uridine disphosphate (UDP)-conjugated amino sugars including UDP-*N*-acetyl glucosamine (UDP-GlcNAc), UDP-glucuronic acid, and UDP-D galacturonate (23). Of these metabolites, UDP-GlcNAc is a rate-limiting substrate of the *O*-GlcNAc transferase (OGT) central to the hexosamine biosynthetic pathway (HBP), a pathway required for glycoprotein formation (26). These findings suggest that RSV replication perturbs cellular glycosylation pathways potentially affecting viral glycoprotein synthesis as well as native protein folding and extracellular matrix (ECM) secretion (27). The role of the HBP in cellular response to RSV infection is unknown.

In this study, we observe that RSV infection induces accumulation of glycosylated proteins in the endoplasmic reticulum (ER) and Golgi complex of SAECs, mediated by increased expression of rate-limiting UDP synthetic enzymes. Understanding that the HBP is a homeostatic response to ER stress, activated by the unfolded protein response (UPR), we explored the effects and consequences of paramyxovirus infection on the UPR. We find that RSV activates rapid formation of spliced X-box binding protein 1 (XBP1s) downstream of inositol-requiring enzyme 1α (IRE1α), in parallel with processing activating transcription factor 6 (ATF6). Our data indicate that inositol-requiring enzyme 1α (IRE1α)-XBP1s is an autoregulated pathway that activates the expression of central EMT regulators. RSV enhances XBP1 binding to the *IL6*, *SNAI1*, and *MMP9* promoters and the intragenic superenhancer of *GFPT2*, an event required for RNA polymerase II engagement. In parallel, murine respirovirus also induces mucosal IL6 and SNAI1 upregulation in mouse lung in an IRE1α-dependent manner, whose expression persists after viral clearance. These data indicate that the UPR is central to metabolic adaptation of glycoprotein metabolism in paramyxovirus infection. We further establish that the UPR mediates paramyxovirus-induced mesenchymal transition and ECM remodeling with potential implications in chronic airway disease.

## MATERIALS AND METHODS

### Human Small Airway Epithelial Cell Culture and Treatment

Human small airway epithelial cells (hSAECs) are immortalized primary human small airway epithelial cells by CDK4/telomerase (hTERT) (28) that undergo growth factor-induced cell-state transition (22) and exhibit RSV-induced genomic and proteomic signatures representative of primary cells (20). hSAECs were grown in SAGM small airway epithelial cell growth medium (Lonza, Walkersville, MD) in humidified incubator with 5% CO_2_. Primary human small airway epithelial cells purchased from American Type Culture Collection (ATCC) (PCS-301-010, at *passage 2*) were cultured in Airway Cell Basal Medium (PCS-300-030, ATCC) supplemented with Bronchial Epithelial Growth kit (PCS-300-040, ATCC) in humidified incubator with 5% CO_2_, and experiments were conducted at *passage 4.* The human RSV long strain was grown in Hep-2 cells, prepared by sucrose cushion purification and titer determined by a methylcellulose plaque assay (29, 30). Viral pools were aliquoted, quick-frozen on dry ice-ethanol, and stored at −70°C until they were used. The selective IRE1α RNAse inhibitor KIRA8 (MedChemExpress, NJ) (31) and the ATF6 processing inhibitor ceapin-A7 (Sigma) (21) were applied to the cells 2 h before RSV infection.

shRNA silencing was performed using lentivirus transduction. For XBP1 or IRE1, five Sigma Mission shRNA lentiviral vectors were generated and populations of transduced hSAECs were selected in 2 µg/mL puromycin. A nontargeting luciferase Sigma Mission shRNA lentiviral vector (Sigma, Cat. No. SHC007) was used as negative control. Gene knock-down efficiencies of the shRNAs were assessed by quantitative reverse transcription polymerase chain reaction (Q-RT-PCR) in the absence or presence of RSV. The target sequences of the shRNAs used for functional studies were: XBP1, 5′- GCCTGTCTGTACTTCATTCAA-3′; IRE1, 5′- GCAGGACATCTGGTATGTTAT-3′.

### RNA Isolation and Q-RT-PCR

Total cellular RNA was isolated using RNeasy kit with on-column DNase digestion (Qiagen) from cultured cells or mouse lung tissue following tissue homogenization using VWR Mini Bead Mill Homogenizer (VWR, PA) and subsequently QIAshredder (Qiagen). Synthesis of complementary DNAs (cDNAs) was done with First Strand cDNA Synthesis kit (Thermo Fisher Scientific). Duplicate Q-RT-PCR assays were performed for each sample in 20 μL PCR reaction using SYBR Green Master mix (Bio-Rad) and gene-specific primers (Table 1) at 500 nM and cDNAs equivalent to 25 ng of total RNA. The PCR was carried out on AriaMx Real-Time PCR System (Agilent, CA) with 40 cycles of 15 s at 95°C and 30 s at 60°C. Data are presented as fold change of mRNA using the ΔΔC_t_ method (32) with cyclophilin A (PPIA) gene as internal control. Among different housekeeping genes, PPIA gene expression is relatively stable under inflammatory stimuli (33).

**Table 1.** Quantitative RT-PCR primers

Gene	Forward	Reverse
hXBP1s	5′-GCTGAGTCCGCAGCAGG-3′	5′-CTCTGGGGAAGGGCATTTGA-3′
hXBP1us	5′-AAGCCAAGGGGAATGAAGTGA-3′	5′-GCCAGAATCCATGGGGAGATG-3′
hXBP1u	5′-ACTCAGACTACGTGCACCTCT-3′	5′-CTGGGTCCAAGTTGTCCAGAA-3′
mXBP1s	5′-GCTGAGTCCGCAGCAGG-3′	5′-TAGCAGACTCTGGGGAAGGA-3′
hIRE1	5′-TGTGTCAACGCTGGATGGAA-3′	5′-TCCACATGTGTTGGGACCTG-3′
hBip	5′-ACTCCTGAAGGGGAACGTCT-3′	5′-CCACCTTGAACGGCAAGAAC-3′
hATF6	5′-CCGTATTCTTCAGGGTGCTCT-3′	5′-AGCTCACTCCCTGAGTTCCTG-3′
hCHOP	5′-CTTCACCACTCTTGACCCTG-3′	5′-TCCTGGTTCTCCCTTGGTCT-3′
hPERK	5′-CTCAGCGACGCGAGTACC-3′	5′-CGGTCGCAACTCTGTCTCAT-3′
hGRP94	5′-GGAGAGTCGTGAAGCAGTTGA-3′	5′-GTTGCCAGACCATCCGTACT-3′
hCRT	5′-GGCAGATCGACAACCCAGAT-3′	5′-GATGGTGCCAGACTTGACCT-3′
hERp72	5′-CTCCAATTCCCCTGGCAAAG-3′	5′-TTTTCTCGTGGGCCGTTGTAG-3′
hP5	5′-GTGAGCAAGGCATCAACGAG-3′	5′-AGGCTCTCTCTCAACGATGG-3′
hPPIB	5′-GGCCCAAAGTCACCGTCAAG-3′	5′-CCGCCCTGGATCATGAAGTC-3′
hPPIC	5′-GACGGCAAACATGTGGTGTT-3′	5′-GACGGTCATGCCCATCAGTT-3′
hHERP	5′-ACTCCTCCCTGAGCAGATTC-3′	5′-AACGTCAGGAGGAGGACCAT-3′
hHRD1	5′-TCCAGGCCTTTGTCCTTGTC-3′	5′-GGGCTGAAGTCATCCCGAAA-3′
hIL6	5′-TGCAATAACCACCCCTGACC-3′	5′-GTGCCCATGCTACATTTGCC-3′
mIL6	5′-CCCCAATTTCCAATGCTCTCC-3′	5′-CGCACTAGGTTTGCCGAGTA-3′
hTGFB1	5′-CTGGACACGCAGTACAGCAA-3′	5′-CGCACGATCATGTTGGACAG-3′
hSNAI1	5′-CGGAAGCCTAACTACAGCGA-3′	5′-GCCAGGACAGAGTCCCAGAT-3′
mSNAI1	5′-CTCCAAACCCACTCGGATGT-3′	5′-AGCCAGACTCTTGGTGCTTG-3′
hZEB1	5′-CACTGCCCAGTTACCCACAA-3′	5′-CAGGGCTGACCGTAGTTGAG-3′
hTWIST	5′-GCTGAGCAAGATTCAGACCCT-3′	5′-CTGCAGCTTGCCATCTTGGA-3′
hFN1	5′-GTACGGCCACCAAGAAGTGA-3′	5′-TGAGACCCAGGAGACCACAA-3′
hCol1A1	5′-GGACACAGAGGTTTCAGTGGT-3′	5′-AGTAGCACCATCATTTCCACGA-3′
hVIM	5′-TGGACCAGCTAACCAACGAC-3′	5′-GCCAGAGACGCATTGTCAAC-3′
hMMP9	5′-CAGTCCACCCTTGTGCTCTT-3′	5′-CGACTCTCCACGCATCTCTG-3′
		
hCDH1	5′-GTGCCTGAGAACGAGGCTAA-3′	5′-TCAAAATCCAAGCCCTTTGCTG-3′
hGFPT1	5′-CTGAGATTGGTGTGGCCAGT-3′	5′-GGCAGCCGTTTCAATCCAAG-3′
hGFPT2	5′-GGGCATCCTGAGCGTGATTC-3′	5′-CCATGTAGCATCCCTGCTGT-3′
mGFPT2	5′-AGAGATCATCCGTGGCCTCA-3′	5′-ATATCCCCGTCCCATCACGA-3′
hDPAGT1	5′-GCAGATCCCAGAATCCCAGG-3′	5′-GCAAGGAGGGCACCTATCAG-3′
hSTT3A	5′-GGCTGGATTGTTGACCTCCTG-3′	5′-CAGCAAACAGACGAGTGGAGA-3′
RSVN	5′-AAGGGATTTTTGCAGGATTGTTT-3′	5′-TCCCCACCGTAACATCACTTG-3′
Sendai virus P	5′-CAAAAGTGAGGGCGAAGGAGAA-3′	5′-CGCCCAGATCCTGAGATACAGA-3′
hPPIA	5′-CGCGTCTCCTTTGAGCTGTT-3′	5′-CCATAGATGGACTTGCCACCA-3′
mPPIA	5′-GTCAACCCCACCGTGTTCTT-3′	5′-ACCACCCTGGCACATGAATC-3′

h, human; m, mouse.

### Immunofluorescence Microscopy

hSAECs were plated on 0.1% gelatin-coated glass coverslips. After treatment, cells were fixed with 4% paraformaldehyde (10 min), permeablized with 0.1% Triton X-100 (10 min), blocked with 5% goat serum in PBS (2 h), and incubated with primary antibody in blocking buffer overnight at 4°C. Primary antibodies used were anti-ATF6 (Cat. No. 24169-1-AP at VWR, 1:50 dilution), SNAI1 (Cat. No. 3895S at Cell Signaling, 1:50 dilution), glutamine-fructose-6-phosphate transaminase 2 (GFPT2) (Abcam, Cat. No. ab190966 at Abcam, 1:100 dilution), VIM (Cell Signaling, Cat. No. 5741S, 1:100 dilution), IRE1α (Abcam Cat. No. ab37073, 1:50 dilution), Calnexin (Abcam Cat. No. ab22595, 1:1,000 dilution), and GOLG2a/GM130 (Abcam Cat. No. ab52649, 1:200 dilution). After washing in PBS, cells were stained for 1 h with Alexa Fluor-conjugated goat secondary antibody, washed in PBS, and mounted using ProLong Diamond Antifade Mountant with 4′,6-diamidino-2-phenylindole (DAPI, Molecular Probes). For staining of glycosylated proteins, following blocking with 5% goat serum or primary antibody incubation (such as anticalnexin or GM130), cells were incubated for 1 h at room temperature with 20 μg/mL FITC-WGA (Sigma) before washing and mounted with ProLong Diamond Antifade Mountant with DAPI. The cells were visualized with Nikon A1RS confocal microscope. Quantitation was done by FIJI. Specifically, three independent experiments were conducted for each treatment. For the quantitation of total cellular protein levels, three fields each containing 25 cells were randomly selected from each experiment and quantitated by FIJI and a mean value of fluorescence intensity [arbitrary units (AU)] per cell was obtained. The background fluorescence was subtracted using control IgG staining.

For formalin-fixed paraffin-embedded (FFPE) mouse lung tissues, following standard deparaffinization and rehydration steps in xylene and reducing concentrations of ethanol, the tissue slides were heated at 95°C for 20 min in 10 mM citrate buffer (pH 6.0) for antigen retrieval. The slides were blocked in 10% goat serum plus mouse on mouse Ig blocking reagent (MOM, Vector, Canada) in Tris-buffered saline (TBS) overnight at 4°C and incubated with SNAI1 antibody (Cat. No. 3895S at Cell Signaling, 1:100 dilution) or control IgG at 4°C overnight afterwards. After washing in TBS-T (0.05% Tween 20), the slides were incubated with Alexa Fluor-conjugated goat secondary antibody for 1 h, washed, and mounted with ProLong Diamond Antifade Mountant with DAPI. The slides were visualized with Nikon A1RS confocal microscope and quantitated by FIJI.

### Immunoblot Analysis

Treated cells were lysed in a phosphoprotein lysis buffer (10 mM Tris at pH 7.5, 100 mM NaCl, 1 mM EDTA, 1 mM EGTA, 0.1% SDS, 1% Triton X-100, 0.5% sodium deoxycholate, 20 mM sodium pyrophosphate, 1 mM β-glycerol phosphate, 10% glycerol, 2 mM activated sodium orthovanadate, and 20 to 50 mM sodium fluoride plus a protease inhibitor cocktail); 50 µg of total protein was used for SDS-PAGE and immunoblot analysis. TATA-box binding protein (TBP) was used as the loading control.

### Quantification of Cellular UDP-GlcNAc

UDP-GlcNAc in treated hSAECs was quantified with LC-SRM-MS as previously described (27).

Briefly, 1 × 10^6^ cells were homogenized in 0.7 M perchloric acid and neutralized supernatants were desalted and directly analyzed by LC-SRM-MS. LC-SRM-MS analysis was performed with a TSQ Vantage triple quadrupole mass spectrometer equipped with a nanospray source (Thermo Fisher Scientific, San Jose, CA). All acquisition methods used the following parameters: 2100 V ion spray voltage, a 275°C ion transferring tube temperature, a collision-activated dissociation pressure at 1.5 mTorr, and the S-lens voltage used the values in S-lens table generated during MS calibration.

### Two-Step Chromatin IP Quantitative Genomic PCR

Protein-protein cross linking was performed with DSG (2 mM, 45 min at 22°C) followed by protein-DNA cross linking with formaldehyde (34). Equal amounts of sheared chromatin were immunoprecipitated (IPed) overnight at 4°C. IPs were collected with 40 μL protein-A magnetic beads (Dynal Inc.), washed, and eluted in 250 µL elution buffer for 15 min at 65°C. Gene enrichment was determined by Q-gPCR using SYBR Green Master mix (Bio-Rad) and region-specific PCR primers (Table 2) at 500 nM. The fold change of specific genomic DNA in each two-step chromatin IP (XChIP) relative to control cells was calculated using the ΔΔC_t_ method (35) and normalized to the absolute amounts of input DNA for each XChIP (35). The ChIP-grade antibodies used in this study are anti-XBP1 (Abcam, Cat. No. ab37152) and anti-RNA Pol II (Abcam, Cat. No. ab26721).

**Table 2.** Q-gPCR primers for XChIP assay

Genic Region	Forward	Reverse
hIL6 promoter	5′-TGGCACAGAGAGCAAAGTCC-3′	5′-AGGTGGAGTGTGTGACTCCT-3′
hSNAI1 promoter	5′-GATGTGCGTTTCCCTCGTCA-3′	5′-CCGGGACACCTGACCTTCC-3′
hSNAI1 3′-UTR	5′-AGCCCAGGCAGCTATTTCAG-3′	5′-CTGGGAGACACATCGGTCAG-3′
hGFPT2 enhancer	5′-GGAGTTGGGACGGAAAGTCA-3′	5′-GAAGCTCACCCTTGCCACTA-3′
hXBP1 promoter	5′-ATCCGTTTGTGGAGGACACG-3′	5′-CGTTTCAGGACCGTGGCTAT-3′
hMMP9 promoter	5′-GAGAGAGGAGGAGGTGGTGT-3′	5′-TTGACAGGCAAGTGCTGACT-3′

h, human; Q-gPCR, quantitative genomic PCR.

### Murine Respirovirus (Sendai virus) Infection

Animal experiments were performed according to the NIH Guide for Care and Use of Experimental Animals and approved by the University of Wisconsin at Madison Institutional Animal Care and Use Committee (Approval No. 1312058 A). Wild-type 7-wk-old C57BL/J6 black mice (both sexes) were administered Sendai Virus (SeV, 10^4^ PFU, Cantell Strain, ATCC) or vehicle (PBS) via the intranasal route. Randomly selected mice were then treated every day with an IRE1α inhibitor (KIRA8, 50 mg/kg/day; MedChemExpress, NJ) for 3 days via the intraperitoneal route starting 24 h after SeV infection. KIRA8 solution at 6 mg/mL was prepared using 10% DMSO-90% corn oil formula and maintained at 37°C for smooth injection. In a separate study, wild-type 7-wk-old C57BL/J6 black mice were administered Sendai Virus (SeV, 10^4^ PFU) or vehicle (PBS) and euthanized at *day 7*. In both studies, lung tissues were collected for preparing FFPE tissue slides for immunofluorescence staining and Q-RT-PCR analysis. Bronchoalveolar lavage fluid (BALF) was also collected for proteomic analysis.

### Trypsin Digestion of BALF Proteins

Eighty microliters of BALF were reduced with 10 mM dithiothreitol (DTT) and were first digested with LysC-trypsin (Promega) followed by trypsin (Promega). Desalted peptides were dried and analyzed by NanoLC-MS/MS.

### NanoLC−MS/MS Analysis

The desalted peptides were separated with a linear gradient of 5%–35% buffer B (100% acetonitrile in 0.1% formic acid) on a C18 reversed-phase column. Mass spectrometry (MS) data were acquired using a data-dependent Top15 method dynamically choosing the most abundant precursor ions from the survey scan (400–1,400 *m/z*) using HCD fragmentation. Survey scans were acquired at a resolution of 70,000 at *m/z* 400. The isolation window was set to 3 Da and fragmented with normalized collision energies of 27. The maximum ion injection times for the survey scan and tandem MS (MS/MS) scans were 20 and 60 ms, respectively, and the ion target values were set to 1E6 and 1e5, respectively. Data were acquired using Xcalibur software.

### Protein Identification

Mass spectra were analyzed using label-free algorithms in MaxQuant software v. 1.5.2.8 using the Andromeda search engine. We required at least one “razor peptide” for quantification. The required false positive rate for identification was set to 1% at the peptide level and 1% at the protein level and the minimum required peptide length was set to six amino acids.

### RSV Infectivity Assay

RSV infectivity was measured by infecting confluent cells with ∼0.5 multiplicity of infection (MOI) of sucrose-purified virus in 24-well tissue culture plate as described previously (36). Briefly, 3 h after adding virus, the cells were washed for three times in growth medium and further cultured for 8, 16, or 24 h. Cells were washed for three times in PBS, fixed in cold methanol:acetone (1:1) at −20°C for 5 min, blocked in PBS + 5% FBS for 2 h, and incubated with goat polyclonal anti-RSV antibody (Cat. No. ab20745 at Abcam, 1:1,000 dilution) at 4°C overnight, followed by washing in PBS and then incubated at RT for 1 h with anti-goat secondary antibody conjugated to β-galactosidase (Abcam, Cat. No. ab136712, 1:2,000 dilution). After washing in PBS, X-Gal substrate (1 mg/mL 5-bromo-4-chloro-3-indoyl-β-galactopyranoside in PBS containing 3 mM potassium ferricyanide, 3 mM potassium ferrocyanide, and 1 mM magnesium chloride) was added and incubated at 37°C for 4–8 h until significant insoluble blue precipitate was formed in the cells. After washing in PBS, the cells were mounted with 70% glycerol. Blue cells were manually counted as RSV focus-forming units (FFU) per field by light microscopy. To examine the effect of KIRA8 on RSV infectivity, confluent hSAECs were pretreated with 10 µM KIRA8 or DMSO for 2 h before addition of RSV.

### Statistical Analyses

Statistical analyses were performed with GraphPad Prism 9 (GraphPad Software, San Diego, CA). Results are expressed as means ± ranges, with *n* representing the number of samples per group. Normality and equal variance tests were performed to determine appropriate application of parametric statistical analyses. If the normality and equal variance tests were passed, differences between groups were tested using ANOVA for repeated measurements, followed by post hoc Tukey’s comparison. Nonparametric comparisons were made by Kruskal–Wallis test. *P* values < 0.05 were considered to be statistically significant.

## RESULTS

### RSV Infection Activates the HBP

To investigate the effect of RSV on UDP-GlcNAc metabolism, we focused our experiments on telomerase-immortalized hSAECs. hSAECs are derived from primary human small airway epithelial cells, maintain representative genomic and proteomic signatures (20), replicate RSV (37), produce pathogenic mucin- and T-helper-2 lymphocyte-activating cytokines that mediate disease (20), and most importantly, exhibit inducible cell state changes (22, 38, 39) without exhibiting cellular senescence.

**Figure 1.**
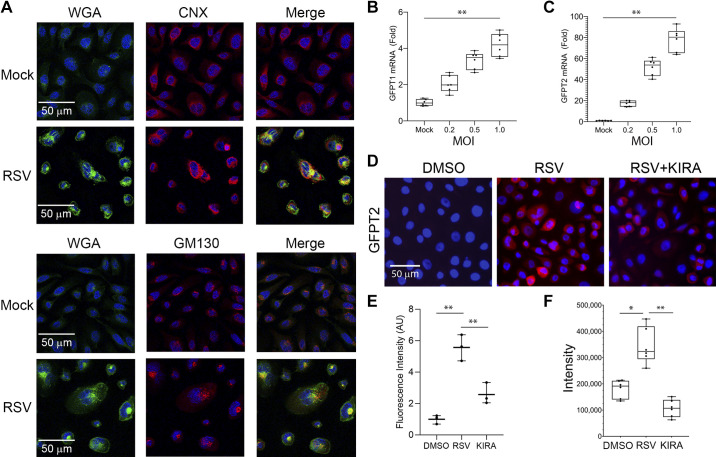
RSV infection induces glycoprotein accumulation and the HBP. *A*: hSAECs were mock-infected or infected with sucrose-purified RSV (MOI = 1.0) for 24 h, fixed and stained for *N*-acetylglucosamino-peptide accumulation using fluorescein-conjugated wheat germ agglutinin (FITC-WGA, green). *Top*: calnexin (CNX) was separately labeled by immunofluorescence staining as an indicator of ER localization (red). The nucleus was stained by DAPI (blue). The merged images indicate substantial overlap of WGA-stained glycoproteins and CNX. Scale bar in microns shown. *Bottom*: GOLG2A/GM130 was labeled as an indicator of Golgi localization. Note the intense perinuclear localization of the RSV-induced WGA staining and the ER (CNX). *B* and *C*: Q-RT-PCR for *GFPT1* and *GFPT2*. hSAECs were infected by RSV for 24 h at different MOI as indicated. The box is 25%–75% interquartile ranges, with means ± ranges. Symbols are individual data points from three independent experiments. **P < 0.01 for multivariate gene expression by MOI. *D*: immunofluorescence microscopy for GFPT2. hSAECs were mock-infected (DMSO) or infected by 1.0 MOI RSV for 24 h in the presence or absence of 10 µM KIRA8 (KIRA) as shown. The nucleus was stained by DAPI (blue). *E*: quantitation of GFPT2 staining in *D* by FIJI. Fold change relative to mock infection (DMSO) was expressed. The box is 25%–75% interquartile ranges, with means ± ranges; symbols are all data points from *n* = 3 independent experiments. **P < 0.01, ANOVA. *F*: quantitation of UDP-GlcNAc. hSAECs were mock-infected (DMSO) or infected by 1.0 MOI RSV for 24 h in the presence or absence of 10 µM KIRA8 (KIRA). Cellular UDP-GlcNAc was quantitated by LC-MS in *n* = 3 independent experiments. Note that RSV induction of UDP-GlcNAc was completely abolished by KIRA8 (*P* < 0.01, ANOVA; pairwise comparison *P < 0.05 and **P < 0.01, post hoc Tukey’s comparison). GFPT2, glutamine-fructose-6-phosphate transaminase 2; HBP, hexosamine biosynthetic pathway; hSAECs, human small airway epithelial cells; MOI, multiplicity of infection; Q-RT-PCR, quantitative reverse transcription polymerase chain reaction; RSV, respiratory syncytial virus; UDP-GlcNAc, UDP-*N*-acetyl glucosamine.

To confirm whether RSV infection activates the HBP, we examined changes in the cellular *N*-glycosylated protein distribution. hSAECs were infected with sucrose-purified RSV [multiplicity of infection (MOI) =1] for 24 h, fixed, and stained for *N*-acetylglucosamino-peptides using fluorescein-conjugated wheat germ agglutinin (FITC-WGA). In confocal microscopy, we observed a substantial accumulation of WGA staining in the perinuclear region of RSV-infected cells and in punctate regions throughout the nucleus (Fig. 1*A*). These areas of *N*-glycosylation accumulation colocalize with calnexin (CNX)-enriched endoplasmic reticulum (ER), indicating that protein *N*-glycosylation in the ER is upregulated in RSV-infected hSAECs (Fig. 1*A*, *top*). By contrast, Goli distribution was determined using GOLGA2/GM130 costaining (Fig. 1*A*, *bottom*). Although *N*-glycosylated species colocalized with the Golgi stain, cytoplasmic colocalization was less than that of the ER, and nuclear retained components of Golgi were detected in response to viral infection. Because the protein *N*-glycosylation requires UDP-GlcNAc, a final metabolic product of HBP (27, 40), the substantial increase in WGA staining suggests that the HBP may be activated by RSV infection.

To further understand the mechanism for enhanced glycosylation, we examined expression of the glucosamine-fructose-6-phosphate aminotransferases (GFPT)-1 and -2 isoforms, representing the rate-limiting enzymes in UDP-GlcNAc production (41). hSAECs were infected with increasing amounts of RSV (0, 0.2, 0.5, and 1.0 MOI) for 24 h and analyzed by Q-RT-PCR. In these experiments, relative changes in gene expression were normalized using expression of *PPIA*, a gene that is inert to RSV infection (Supplemental Fig. S1; all Supplemental material is available at https://doi.org/10.6084/m9.figshare.14912631.v1). We observed an MOI-dependent increase in *GFPT1* expression, peaking at 4.2 ± 0.67-fold (*P* = 3.52E-07) relative to uninfected cells at an MOI =1.0 (Fig. 1*B*). Compared with the expression of *GFPT1*, *GFPT2* was much more inducible, with a 77.8 ± 11.2-fold (*P* = 1.13E-08) induction at an MOI of 1.0 (Fig. 1*C*).

In response to TGFβ stimulation, the HBP pathway is induced at least partially via the IRE1α-XBP1s arm of the unfolded protein response (UPR) pathway (14). To further characterize the effect of RSV infection on GFPT2 and test whether GFPT2 expression was IRE1α dependent, we performed immunofluorescence microscopy for GFPT2 in RSV-infected cells with or without the IRE1α RNAase inhibitor, KIRA8. KIRA8 is a highly selective inhibitor that allosterically attenuates IRE1α RNase activity with nM affinity (31). We observed that RSV infection induced 5.5 ± 1.0-fold (*P* = 8.27E-04) increase in cytoplasmic GFPT2 immunostaining, whose induction was substantially reduced by KIRA8 (*P* < 0.01, ANOVA, Fig. 1, *D* and *E*).

Next, to confirm RSV activation of the HBP and the regulatory role of IRE1α pathway, we measured the levels of UDP-GlcNAc in RSV-infected hSAECs. RSV infection increased the production of UDP-GlcNAc by 1.8-fold (Fig. 1*F*). This increase in intracellular UDP-GlcNAc was completely abolished by the treatment with KIRA8. Together, these data indicate that RSV infection induces *N*-linked glycoprotein accumulation within the ER, and expression of rate-limiting biosynthetic enzymes of UDP-GlcNAc in an IRE1α-dependent manner.

### RSV Is an Activator of the IRE1α-XBP1 and ATF6 Arms of the UPR

Based on our earlier studies showing that the UPR is upstream of the HBP in TGFβ-induced EMT (27) and the dependence of GFPT2/UDP GlcNAc on IRE1α activity (Fig. 1), we were prompted to directly examine whether RSV replication activates major transcription regulatory arms of the UPR. Of the UPR pathways, the IRE1α-XBP1 arm is the most conserved from yeast to mammals. IRE1α endoribonuclease and kinase activities are stimulated by dissociation from immunoglobulin binding chaperone (BiP/Grp78) in response to unfolded proteins accumulating in the ER. Upon IRE1α dimerization/oligomerization and autophosphorylation, its intrinsic endoribonucleolytic activity splices a 26 nt sequence from the unspliced (u) *XBP1u* mRNA sequestered in a cotranslational complex, producing a frame-shifted, spliced form of XBP1 (XBP1s) that functions as a transcription factor (42).

**Figure 2.**
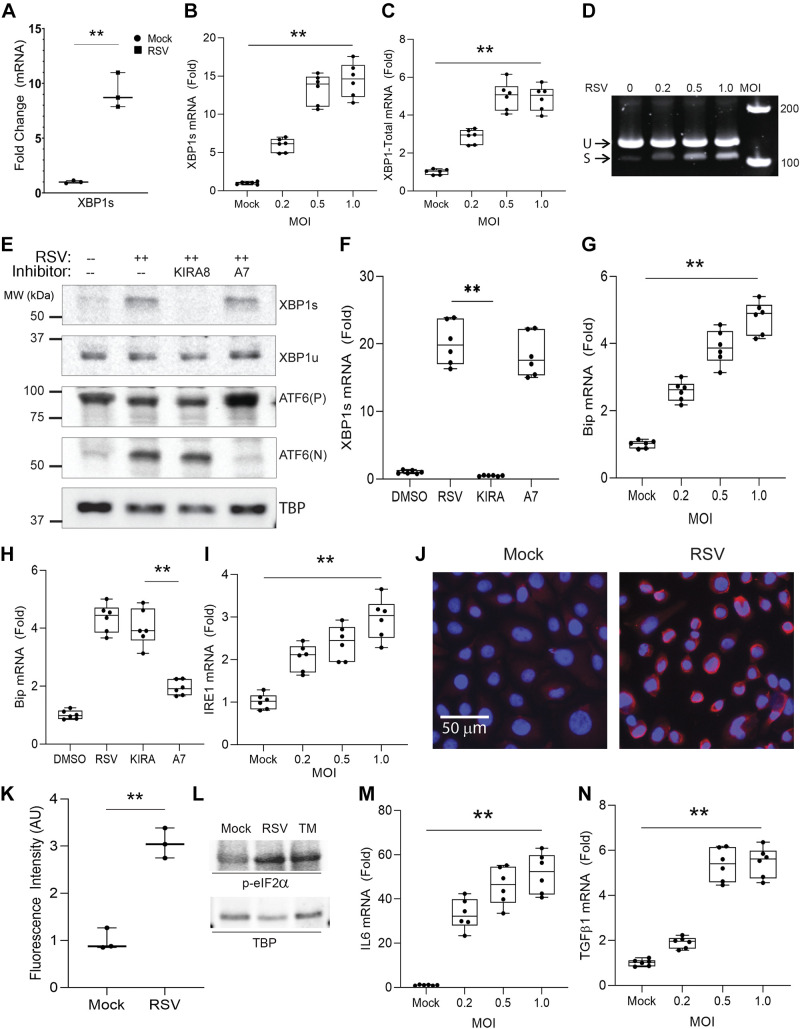
RSV replication activates the IRE1α-XBP1 and ATF6 arms of the UPR. *A:* primary human small airway epithelial cells at *passage 4* were infected with 1.0 MOI RSV for 24 h and Q-RT-PCR was carried out for spliced XBP1 (*XBP1s*). Shown is the fold change of mRNA relative to mock infection in *n* = 3 independent experiments. Telomerase-immortalized hSAECs were infected with different amounts of RSV as indicated for 24 h and Q-RT-PCR was performed for *XBP1s* (*B*), *XBP1*-Total (unspliced + spliced, *C*), *Bip/Grp78* (*G*), *IRE1* (*I*), *IL6* (*M*), and *TGFβ1* (*N*). Fold change of mRNA relative to mock infection is expressed. Boxes are 25%–75% interquartile ranges, with means ± ranges. Symbols are all individual data points from three independent experiments. **P < 0.01 for gene expression by MOI using ANOVA. *D:* Q-RT-PCR product for total *XBP1* mRNA was fractionated by polyacrylamide gel electrophoresis with ethidium bromide staining. Migration of unspliced (U) and spliced (S) transcripts is shown with DNA ladder (in bp at right). *E:* Western blot for XBP1 and ATF6. hSAECs were infected by RSV for 24 h at an MOI of 1.0 in the presence or absence of 10 µM KIRA8 (KIRA) or ceapin-A7 (A7). TATA-box binding protein (TBP) was probed as a loading control. MW, molecular weight standards in kDa. Q-RT-PCR for *XBP1s* (*F*) or *Bip/Grp78* (*H*) mRNA. hSAECs were treated as in *E.* Bars represent fold induction of mRNA relative to mock infection (DMSO) with means ± ranges. Symbols are all data points from three independent experiments. **P < 0.01 for post hoc comparison. *J*: immunofluorescence microscopy for IRE1α. hSAECs were infected by 1.0 MOI RSV for 24 h. The nucleus was stained by DAPI (blue). *K*: the fluorescence intensity of IRE1α staining in *J* was quantitated by FIJI. Fold change relative to mock infection was expressed. (*L*) Western blot. *Top*: phosphorylated eIF2α abundance for Mock, RSV (MOI = 1, 24 h), or tunicamycin (TM, 5 µg/mL, 8 h) treatment. *Bottom*: TBP as internal control. hSAECs, human small airway epithelial cells; MOI, multiplicity of infection; Q-RT-PCR, quantitative reverse transcription polymerase chain reaction; RSV, respiratory syncytial virus; UPR, unfolded protein response.

To confirm RSV-induced activation of the XBP1 pathway, we first measured production of *XBP1s* mRNA in primary human small airway epithelial cells using a splice-form selective Q-RT-PCR assay (Table 1). We observed that RSV infection induced a 9.2 ± 1.6-fold (*P* = 9.26E-04) increase in *XBP1s* (MOI = 1, 24 h; Fig. 2*A*).

To explore activation of XBP1 more thoroughly, we designed Q-RT-PCR assays to quantitate total (spliced + unspliced) *XBP1* (*XBP1*-Total) along with the spliced *XBP1s* mRNAs. In telomerase-immortalized hSAECs, we found that RSV infection induced an MOI-dependent increase in *XBP1*-Total and *XBP1s*, peaking at 14.53 ± 2.2-fold (*P* = 4.53E-08) for *XBP1s* and 5.49 ± 0.7-fold (*P* = 6.61E-08) for *XBP1*-Total at an MOI of 1 (Fig. 2, *B* and *C*). Polyacrylamide gel analysis of the *XBP1*-Total Q-RT-PCR end-point product revealed both unspliced (XBP1u) and spliced (XBP1s) isoforms and suggested that the induced spliced form is a relatively small fraction of its precursor (Fig. 2*D*). We further designed XBP1u isoform-selective Q-RT-PCR primers and observed similar RSV response in *XBP1u* compared with *XBP1-Total* (Supplemental Fig. S3). The significant upregulation of the *XBP1 Total* mRNA could serve to sustain IRE1-mediated XBP1s production in RSV infection.

To confirm that RSV induces expression of XBP1s protein, Western immunoblot assays were performed. We observed an increase in abundance of the ∼55 kDa XBP1s isoform in total cell extracts in response to RSV infection (MOI = 1, Fig. 2*E*). The treatment with the selective allosteric IRE1α ribonuclease inhibitor, KIRA8, completely abolished XBP1s formation at both protein (Fig. 2*E*) and mRNA (Fig. 2*F*) levels, confirming both the accuracy of our assays and the specificity of the inhibitor.

A separate arm of the UPR engaged in gene transactivation, ATF6(N) (p50), is activated in a distinct manner by the site-1/2 proteases (S1P/S2P)-mediated cleavage of ATF6(P) (p90) following its Golgi translocation from the ER in response to UPR-activating ER stress, resulting in the production of the nucleus-localized active transcription factor ATF6(N) (p50). Ceapin-A7 blocks ATF6 transport to the Golgi for proteolytic processing (21). Western blot confirmed that RSV infection also induced the 50 kDa ATF6(N) protein; ATF6(N) induction was abolished by treatment with ceapin-A7 but unaffected by KIRA8 (Fig. 2*E*). We further noted that the inhibition of ATF6(N) production was accompanied by increased levels of the p90 ATF6(P) isoform (Fig. 2*E*), suggesting that the ceapin-A7 treatment altered posttranslational ATF6 processing. This conclusion is further confirmed by ATF6 immunofluorescence assay, where RSV significantly induces nuclear ATF6(N), whose induction was completely abolished by ceapin-A7 (Supplemental Fig. S2; note that the antibody only recognizes the activated nuclear form and not the native precursor). To test whether ATF6(N) is functionally active, we examined expression of the chaperone, Bip/Grp78, which is a well-recognized ER stress marker and known to be a direct transcriptional target of ATF6(N) (43). As shown in Fig. 2, *G* and *H*, RSV dose-dependently induced *Bip/Grp78* mRNA, and this induction was effectively suppressed by the selective ATF6 pathway inhibitor ceapin-A7 but not by KIRA8. These data confirm RSV activation of the IRE1α-XBP1s and ATF6 UPR pathways and demonstrate the selectivity of the KIRA8 and ceapin-A7 inhibitors for the IRE1α-XBP1s and S1P/S2P-ATF6(N) pathways, respectively. In addition to *Bip/Grp78*, glucose-regulated protein *(GRP)94* and calreticulin (*CRT*) are solely regulated by ATF6(N) (44), and showed an MOI-dependent significant induction by RSV (Supplemental Fig. S3). Collectively, these data indicate that RSV also induces the ATF6 effector arm of the UPR.

We further noted that both *IRE1α* mRNA and protein levels were upregulated threefold (*P* < 0.01) in response to RSV infection (at MOI 1.0 and 24 h. Fig. 2, *I–K*). Finally, we measured activation of the PERK pathway by phospho-eIF2α in Western blot. Both RSV and tunicamycin (TM) induced a 2–3-fold activation of phospho-eIF2α (Fig. 2*L*). Together, these data indicate that RSV replication activates all three arms of the UPR.

### RSV Primes Expression of Core EMT Regulators and ECM Expression

Previously we found that the UPR propagates EMT by activating autocrine release of epithelial growth factors that function in a paracrine manner (27). To examine whether this process occurs in response to RSV, we measured expression of *IL6* and *TGFβ1* mRNA. We observed a 51.60 ± 9.1-fold (*P* = 9.14E-08) increase in *IL6* mRNA expression and a 5.58 ± 0.7-fold (*P* = 1.96E-08) increase in *TGFβ1* mRNA in RSV-infected cells at an MOI of 1, compared with uninfected cells (Fig. 2, *M* and *N*).

**Figure 3.**
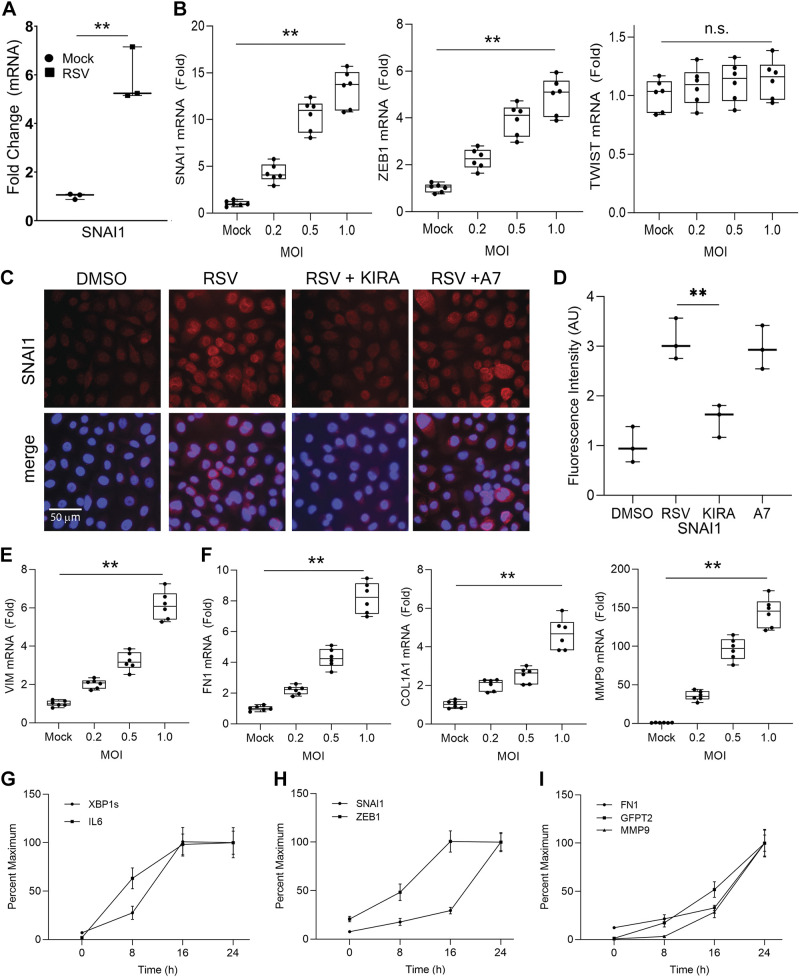
RSV activates EMT. *A:* primary human small airway epithelial cells (hSAECs) at *passage 4* were infected with RSV (MOI = 1) for 24 h and Q-RT-PCR was performed. Shown is fold change of *SNAI1* mRNA relative to mock-infected control in *n* = 3 independent experiments. RSV-induced changes in the expression of the core EMT transcriptional regulators *SNAI1*, *ZEB1*, and *TWIST* (*B*) and the EMT/ECM-related proteins *VIM* (*E*), *FN1*, *COL1A1*, and *MMP9* (*F*) in telomerase-immortalized hSAECS. hSAECs were infected for 24 h by different amounts of RSV as indicated and Q-RT-PCR was carried out to determine the fold change of mRNA relative to mock infection. Boxes are 25%–75% interquartile ranges, with means ± ranges. Symbols are data points from *n* = 3 independent experiments. **P < 0.01 for gene expression by MOI. n.s., not significant. *C*: immunofluorescence microscopy of SNAI1. hSAECs were infected by RSV for 24 h at an MOI of 1.0 in the presence or absence of 10 µM KIRA8 (KIRA) or ceapin-A7 (A7). The nucleus was stained by DAPI (blue). Scale bar in microns shown. *D:* quantitation of SNAI1 staining in *C* by FIJI. Fold change relative to mock infection (DMSO) was expressed. Boxes are 25%–75% interquartile ranges, with means ± ranges. Symbols are quantification from *n* = 3 experiments. *G–I*: temporal expression patterns of the UPR, HBP, and EMT molecules. hSAECs were infected with RSV (MOI = 1.0) and harvested at 0, 8, 16, and 24 h. Relative mRNA levels were determined by Q-RT-PCR. Shown is the percentage of the maximum gene expression at 24 h. Note the maximal expression of *XBP1s* and *IL6* at 16 h, followed by expression of *SNAI1*, *FN1*, *GFPT2*, and *MMP9* that peak at 24 h and later. Bars represent means ± ranges of three independent experiments. **P < 0.01, ANOVA. ECM, extracellular matrix; EMT, epithelial mesenchymal transition; HBP, hexosamine biosynthetic pathway; hSAECs, human small airway epithelial cells; IRE1α-XBP1, inositol-requiring enzyme 1α-X-box binding protein 1; MOI, multiplicity of infection; Q-RT-PCR, quantitative reverse transcription polymerase chain reaction; RSV, respiratory syncytial virus; SNAI1, Snail family transcriptional repressor 1; UPR, unfolded protein response.

We next investigated whether RSV infection would induce EMT in small airway epithelial cells. First, we analyzed the gene expression of core mesenchymal transcription factor SNAI1 in primary human small airway epithelial cells, where we observed RSV increased *SNAI1* mRNA expression by 5.8 ± 1.1-fold (*P* = 1.82E-03) (Fig. 3*A*). To dissect the MOI dependence and kinetics of the RSV-induced EMT network, we examined *SNAI1*, Zinc-finger enhancer binding 1 (*ZEB1*), and *TWIST* expression in telomerase-immortalized hSAECs. Consistent with the findings in the primary human small airway epithelial cells, we observed that *SNAI1* mRNA was upregulated by 13.3 ± 2.0-fold (*P* = 3.76E-08) at an MOI of 1.0 with ZEB1 induced 4.9 ± 0.8-fold (*P* = 3.52E-07) and no significant induction of *TWIST* (Fig. 3*B*). Consistently, SNAI1 protein upregulation in the nucleus and cytoplasm was observed by immunofluorescence microscopy, where a 3.0 ± 0.4-fold (*P* = 6.76E-03) in total cellular SNAI1 content was seen. The induction of SNAI1 was substantially reduced by the IRE1α inhibitor KIRA8, but not by the ATF6 processing inhibitor, ceapin-A7 (Fig. 3, *C* and *D*). We also noted the upregulation of the mesenchymal intermediate filament *VIM* by 6.1 ± 0.7-fold (*P* = 1.39E-08) and the ECM proteins *FN1*, *Col1A1*, and *MMP9* by 8.2 ± 1.0-fold (*P* = 9.18E-09), 5.47 ± 0.8-fold (*P* = 8.43E-07), and 143.60 ± 19.2-fold (*P* = 5.51E-09), respectively, when infected by RSV for 24 h at an MOI of 1.0 (Fig. 3, *E* and *F*). These data contrast with our earlier findings on the TGFβ pathway that IRE1α-XBP1 signaling is upstream of *SNAI1* expression.

### RSV Activation of IRE1α-XBP1s UPR Pathway Precedes That of EMT Program

Detailed time-course studies of TGFβ-induced EMT found that the UPR pathway was activated late in the development of EMT (27). We therefore conducted a series of time-course experiments to examine the temporal relationship between RSV infection, *XBP1s* formation, and EMT. Q-RT-PCR analyses were performed and expressed as percentage of the maximum gene expression at 24 h. At a wide range of MOIs (from 0.2 to 1.0 MOI), *XBP1s* formation was maximal after 16 h of infection and plateaued by 24 h (data only shown for MOI = 1, Fig. 3*G*). *IL6* expression showed a similar rapid kinetic response (Fig. 3*G*). The core EMT regulator *SNAI1* (Fig. 3*H*), the mesenchymal intermediate filament *VIM* (Supplemental Fig. S5), and the ECM remodeling proteins *FN1* (Fig. 3*I*), COL1A1 (Supplemental Fig. S5) and *MMP9* (Fig. 3*I*) as well as the rate-limiting HBP enzyme *GFPT2* (Fig. 3*I*) all showed a delayed expression relative to that of XBP1s. These data indicated that activation of IRE1α-XBP1s arm of the UPR pathway was an early, rapid response to RSV infection and precedes that of the EMT program.

### The HBP and EMT Pathways Are IRE1α-Dependent

**Figure 4.**
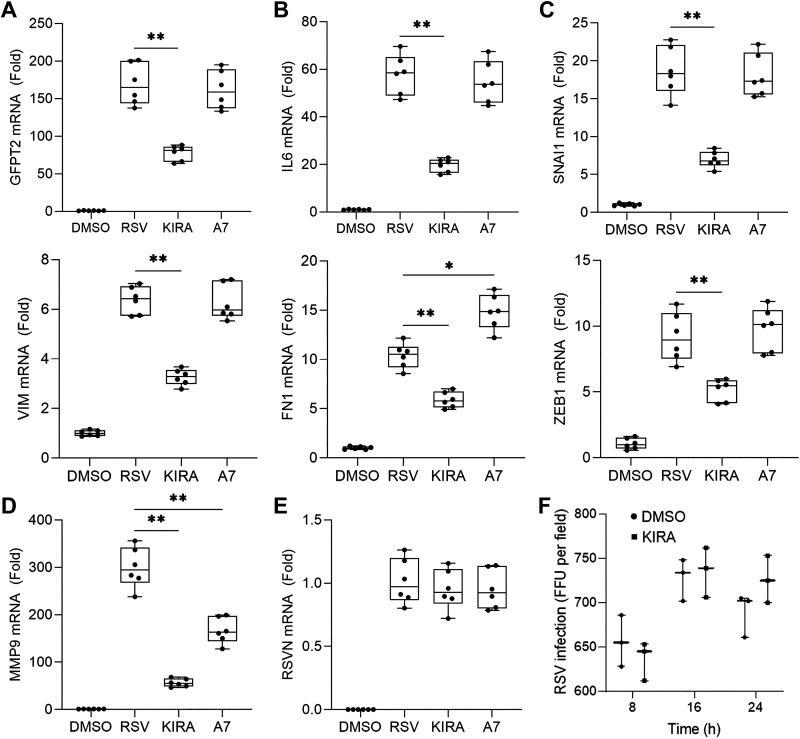
IRE1α-XBP1 pathway regulates HBP and EMT gene networks: IRE1α kinase inhibitor. hSAECs were infected with RSV (MOI = 1.0) for 24 h in the absence (DMSO, solvent carrier) or presence of the IRE1α endoribonuclease inhibitor KIRA8 (KIRA) or the ATF6 inhibitor ceapin-A7 (A7) at 10 µM. Shown is fold change of mRNA relative to mock infection determined by Q-RT-PCR. Bars represent means ± ranges plus all data points of three independent experiments. *GFPT2* (*A*); *IL6* (*B*); *SNAI1, ZEB1, VIM,* and *FN1* (*C*); *MMP9* (*D*); and effect on RSV transcription (*E*). Note the equivalent expression of RSV N transcript between treatments indicates that RSV replication was not significantly affected by either KIRA8 or ceapin-A7. *F*: effect on RSV infectivity. Shown are focus forming units (FFU) determined by colorimetric assay using polyclonal anti-RSV antibodies. *P < 0.05, **P < 0.01, post hoc Tukey’s pairwise comparison. EMT, epithelial mesenchymal transition; HBP, hexosamine biosynthetic pathway; hSAECs, human small airway epithelial cells; IRE1α-XBP1, inositol-requiring enzyme 1α-X-box binding protein 1; Q-RT-PCR, quantitative reverse transcription polymerase chain reaction; RSV, respiratory syncytial virus; ZEB1, zinc finger E-box binding homeobox 1.

We had observed the IRE1α-dependence of RSV induction of HBP (GFPT2 and UDP-GlcNAc, Fig. 1, *D* and *F*) and SNAI1 (Fig. 3, *C* and *D*). To further understand the distinct contribution of IRE1α-XBP1 and ATF6 arms of the UPR to the activation of the HBP and the EMT programs, we infected hSAECs with RSV in the absence or presence of highly selective small molecule inhibitors of the IRE1α RNAase (KIRA8), or ATF6(P) processing (ceapin-A7) as demonstrated earlier (Fig. 2, *E*–*H*). We observed that inhibition of IRE1α-XBP1 pathway preferentially inhibited RSV-induced gene expression of *GFPT2*, *IL6*, and the EMT regulators (*SNAI1, ZEB1*) and mesenchymal (*VIM*) and ECM proteins (*FN1*), but ceapin-A7 did not display significant inhibitory effects on these genes (Fig. 4, *A*–*C*). As seen with SNAI1 (Fig. 3, *C* and *D*) and GFPT2 (Fig. 1, *D* and *E*), the IRE1α-dependence of VIM expression was also confirmed by immunofluorescence microscopy (Supplemental Fig. S4). Consistent with GFPT2’s role as a rate-limiting enzyme of HBP, KIRA8 substantially reduced the RSV-induced glycoprotein accumulation in the perinuclear region of the cells (Supplemental Fig. S6).

By contrast, *MMP9* was inhibited by both inhibitors, indicating that both the IRE1α-XBP1 and ATF6 arms coordinately regulate *MMP9* gene expression, with IRE1α-XBP1 as the more significant regulator (Fig. 4*D*). To control for any potential artifactual effect of the inhibitors on viral infectivity, Q-RT-PCR for RSV N mRNA and RSV infectivity assay (measuring expression of RSV F glycoprotein) was performed. Neither KIRA8 nor ceapin-A7 had a significant effect on RSV transcription (Fig. 4*E*). No significant inhibitory effect on RSV infectivity was observed for KIRA8 at all time points tested (Fig. 4*F*). Taken together with the temporal profiles of XBP1s formation preceding that of the HBP and EMT pathways (Fig. 3, *G*–*I*), these results indicate that RSV-induced HBP and EMT as well as ECM production are primarily downstream of the IRE1α-XPB1 pathway.

### Gene Silencing the IRE1α-XBP1 Pathway

**Figure 5.**
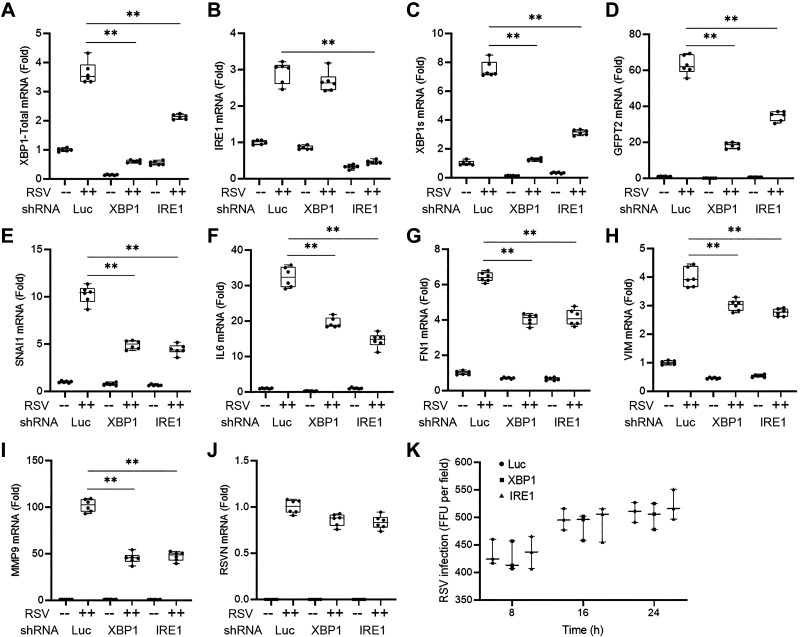
IRE1α-XBP1 pathway regulates HBP and EMT gene networks: shRNA silencing. Q-RT-PCR analysis of hSAECs stably expressing nontargeting shRNA (Luc), IRE1α-targeting shRNA (IRE1), or XBP1-targeting shRNA (XBP1). Cells were mock or RSV-infected (MOI = 1, 24 h). Shown is fold change of mRNA relative to mock infection determined by Q-RT-PCR. Bars represent means ± ranges plus all data points of three independent experiments. **P < 0.01. *A*: *XBP1-*Total; *B*: *IRE1α*; *C*: *XBP1s*; *D*: *GFPT2*; *E*: *SNAI1*; *F*: *IL6*; *G*: *FN1*; *H*: *VIM*; *I*: *MMP9*; *J*: *RSV N* transcription; *K*: RSV infectivity by colorimetric measurement of FFUs. EMT, epithelial mesenchymal transition; HBP, hexosamine biosynthetic pathway; hSAECs, human small airway epithelial cells; IRE1α inositol-requiring enzyme 1α; MOI, multiplicity of infection; Q-RT-PCR, quantitative reverse transcription polymerase chain reaction; RSV, respiratory syncytial virus; SNAI1, Snail family transcriptional repressor 1; XBP1, X-box binding protein 1.

To confirm and extend the results of the small molecule IRE1α inhibitor, we examined the effects of separately silencing XBP1 and IRE1α genes. hSAECs were transduced by lentiviral vectors expressing luciferase (Luc), XBP1, or IRE1α-specific shRNA, and stable populations selected. Cells were then exposed to Mock or RSV infection (MOI = 1, 24 h) and changes in mRNA measured. As shown in Fig. 5, *A* and *B*, in contrast to control knockdown (KD) cells (Luc), both basal and RSV-induced *XBP1* and *IRE1α* expression were effectively silenced. In mock-infected cells, the basal level of *XBP1*-Total mRNA was reduced to 0.14 ± 0.007-fold (*P* = 3.12E-12 for control vs. KD contrast) in XBP1 KD cells (Fig. 5*A*), and the RSV-induced *XBP1* mRNA expression reduced from 3.6 ± 0.37 fold to 0.6 ± 0.049 (*P* = 2.5E-09) in XBP1 KD cells (Fig. 5*A*). Similarly in mock-infected cells, basal *IRE1α* mRNA was reduced to 0.33 ± 0.053-fold (*P* = 9.64E-10) in IRE1α KD relative to control KDs (Fig. 5*B*). And the 2.9 ± 0.3-fold induction of *IRE1α* mRNA in response to RSV infection was dramatically inhibited to 0.45 ± 0.05-fold (*P* = 2.1E-09) in IRE1 KDs (Fig. 5*B*). These data indicate successful silencing of XPB1 and IRE1α.

We noted that silencing of XBP1 had little effect on *IRE1α* mRNA expression (Fig. 5*B*). This result confirms that XBP1s formation is downstream of IRE1α. Importantly, silencing either XBP1 or IRE1α both significantly downregulated the basal and RSV-induced *XBP1s*. In contrast to the robust 7.5 ± 0.56-fold induction of *XBP1s* mRNA by RSV infection in control cells, *XBP1s* induction was reduced to 1.25 ± 0.097-fold (*P* = 1.18E-10) in XBP1 KDs and 3.1 ± 0.18-fold (*P* = 5.6E-09) in IRE1 KDs (Fig. 5*C*). IRE1 silencing significantly inhibited RSV induction of XBP1 mRNA (3.6 ± 0.37-fold induction in control KD vs. 2.13 ± 0.08-fold induction in IRE1 KD, *P* = 2.29E-06) (Fig. 5*A*). This indicates a regulatory role for IRE1α-XBP1s pathway in XBP1 gene expression. Collectively, these data indicate a functional inhibition of XBP1 and IRE1 genes were accomplished.

We noted that XBP1 and IRE1α silencing substantially inhibited expression of *GFPT2* mRNA, confirming our earlier finding that the HBP is dependent on the IRE1α-XBP1 axis (Fig. 5*D*). Compared with control KD with mock infection, RSV induction of *GFPT2* mRNA in control KD cells was 63 ± 5.2-fold (*P* = 5.17E-11), whereas that in XBP1 and IRE1α KD cells were downregulated to 18.3 ± 1.64-fold (*P* = 2.04E-09) and 34.6 ± 2.71-fold (*P* = 3.22E-07), respectively; the relative basal level of GFPT2 was also downregulated dramatically by knock-down of XBP1 (0.12 ± 0.01-fold, *P* = 5.03E-08) and significantly by knockdown of IRE1α (0.64 ± 0.039-fold, *P* = 1.69E-04).

Similarly, RSV-induced expression of the core mesenchymal regulatory genes, SNAI1, IL6, FN1, and VIM, was inhibited as well as the ECM regulator, MMP9 (Fig. 5, *E*–*I*). Specifically, compared with control KD with mock infection, RSV induced *SNAI1, IL6*, *MMP9*, *FN1*, and *VIM* in control KD cells by 10.2 ± 0.93-fold (*P* = 3.31E-10), 32.4 ± 2.9-fold (*P* = 1.35E-10), 101.8 ± 6.81-fold (*P* = 6.07E-12), 6.4 ± 0.27-fold (*P* = 5.95E-13), and 3.99 ± 0.34-fold (*P* = 1.37E-09), respectively; with XBP1 KD, these inductions were downregulated to 4.92 ± 0.46-fold (*P* = 1.91E-07), 19.4 ± 1.43-fold (*P* = 1.92E-06), 45.1 ± 5.64-fold (*P* = 2.26E-08), 4.0 ± 0.3-fold (*P* = 5.23-08), and 3.0 ± 0.2-fold (*P* = 1.31E-04), respectively. And with IRE1 KD, RSV induction of the same genes was downregulated to 4.42 ± 0.52-fold (*P* = 1.09E-07), 14.5 ± 1.99-fold (*P* = 2.11E-07), 47.2 ± 4.97-fold (*P* = 2.06E-08), 4.1 ± 0.46-fold (*P* = 1.05E-06), and 2.8 ± 0.13-fold (*P* = 9.66E-06), respectively. By contrast, transcription of RSV N (Fig. 5*J*) and RSV infectivity (Fig. 5*K*) was not significantly affected by disruption of the IRE1α-XBP1 pathway. These data validate the earlier findings produced by the small molecule IRE1α inhibitor.

### XBP1 Interacts and Recruits RNA Polymerase to the Core EMT and HBP Regulators

**Figure 6.**
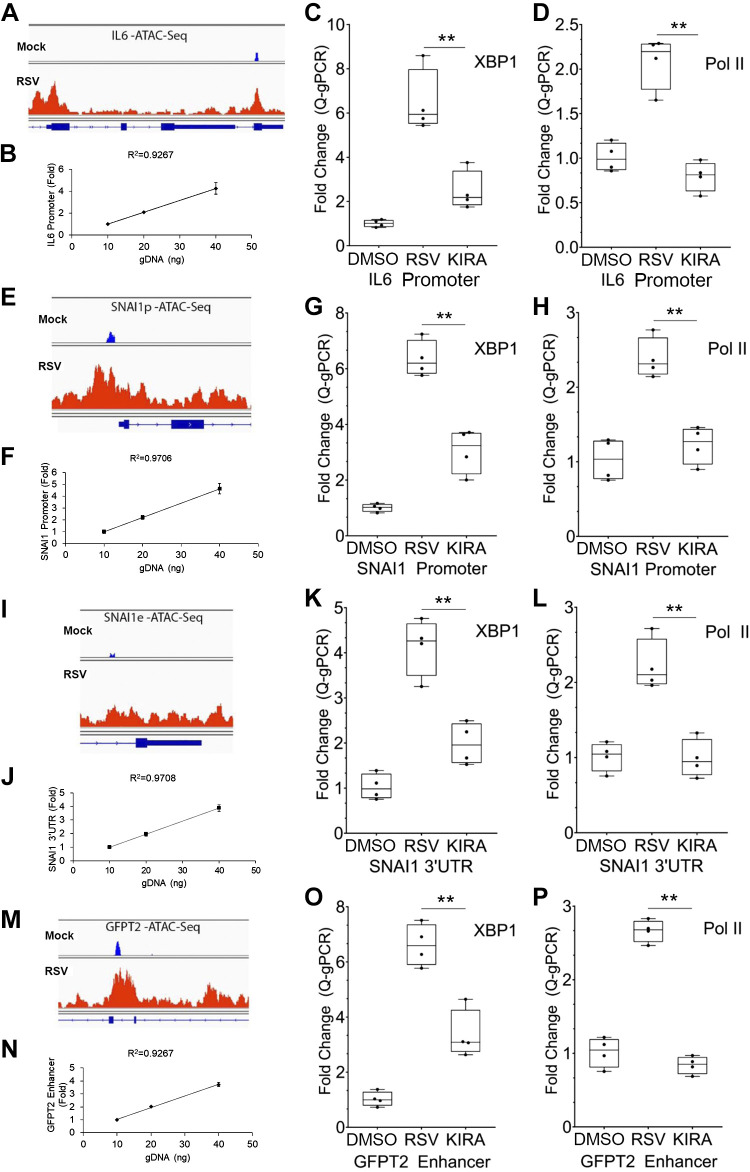
XBP1 is a direct regulator of *IL6*, *SNAI1*, and *GFPT2* genes. *A, E, I,* and *M* are integrated genomics viewer (IGV) of the individual ATAC-seq peaks for uninfected (Mock) and RSV-infected hSAECs. Transposase peaks are indicated by the height of the red graphs and located relative to each gene. Note the dramatic increase in transposase cleavage in the regulatory regions in response to RSV. For the XChIP assays in this study, hSAECs were infected with RSV (MOI = 1.0) for 24 h in the absence or presence of the IRE1α RNase inhibitor KIRA8 at 10 μM (KIRA). *B, F, J,* and *N* are standard curves for the performance of the quantitative genomic PCR (Q-gPCR) amplifying the genomic DNA regions of *IL6* promoter (*B*), *SNAI1* promoter (*F*), *SNAI1* 3′UTR (*J*), and *GFPT2* intragenic superenhancer (*N*) (see the PCR primers in Table 2). Increasing amounts of genomic DNA isolated from hSAECs were applied. Note that each curve has a regression line with *R^2^* > 0.92. *C, G, K,* and *O* are XChIP assays for XBP1 binding to indicated genes. The genomic DNA enrichment by XBP1 XChIP was assayed by Q-gPCR using primers specific for *IL6* promoter (*C*), *SNAI1* promoter (*G*), *SNAI1* 3′UTR (*K*), and *GFPT2* intragenic superenhancer (*O*). Data are calculated as fold change relative to uninfected hSAECs (DMSO) and plotted as means ± ranges plus all data points of duplicate independent immunoprecipitates. *D, H, L*, and *P* are XChIP assays of RNA polymerase II (Pol II) binding to indicated genes. Q-gPCR assays were performed as in *C, G, K*, and *O*. IgG controls of the XChIP had less than 1/10 Q-gPCR signal relative to XChIP with either XBP1 or RNA Pol II antibodies (not shown). **P < 0.01, ANOVA with post hoc Tukey’s comparison. ATAC-seq, assay for transposase-accessible chromatin using sequencing; hSAECs, human small airway epithelial cells; MOI, multiplicity of infection; RSV, respiratory syncytial virus; SNAI1, Snail family transcriptional repressor 1; XBP1, X-box binding protein 1.

XBP1s is a potent transcription factor that directly interacts with regulatory regions of the genome modified by context- and stimulus-dependent interactions with chromatin complexes (45). In a previous study, we identified RSV-inducible domains of open chromatin using Tn transposase cleavage (ATAC)-next-generation sequencing (46). The promoters and/or enhancers of highly inducible cytokines (such as IL6), core EMT regulators (such as SNAI1), and EMT/ECM-related proteins (such as GFPT2 and MMP9) are induced to open chromatin configuration by RSV infection, as visualized by individual tracks in the Integrated Genomic Viewer (Fig. 6, *A*, *E*, *I*, and *M*; Supplemental Fig. S7).

To determine whether RSV-induced XBP1s interacts with these open chromatin domains, we performed chromatin immunoprecipitation (ChIP) using a sequential two-step cross-linking technique that preserves indirect protein interaction by fixation of protein–protein interactions first, followed by protein-DNA cross-linking (35). Mock or RSV-infected hSAECs in the absence or presence of KIRA8 treatment were subjected to two-step ChIP with IgG, anti-XBP1, or anti-RNA Pol II antibodies. The binding of XBP1s and RNA Pol II to *IL6*, *SNAI1*, *GFPT2*, and *MMP9* genes was determined by quantitative genomic PCR (Q-gPCR), which demonstrated linearity over the genomic DNA concentration ranges in the IP (Fig. 6, *B*, *F*, *J*, and *N*; Supplemental Fig. S7). We observed that RSV induced a 6.5 ± 1.4-fold (*P* = 2.81E-04) increase of XBP1 binding to the promoter of *IL6*, and the induction was substantially reduced by treatment with KIRA8 (Fig. 6*C*). Similarly, the binding of RNA polymerase II to the *IL6* promoter was increased in RSV-infected cells by 2.1 ± 0.3-fold (*P* = 7.11E-04), but was blocked by KIRA8 treatment (Fig. 6*D*).

Both the upstream promoter and downstream UTR of the *SNAI1* gene are opened by RSV infection (Fig. 6, *E* and *I*). XChIP confirmed a robust 6.4 ± 0.6-fold (*P* = 3.67E-06) increase of XBP1 binding and a 2.4 ± 0.35-fold (*P* = 4.51E-04) increase in RNA Pol II binding to the *SNAI1* promoter, which was largely inhibited by KIRA8 (Fig. 5, *G* and *H*). A similar pattern was seen for the 3′UTR of *SNAI1* (Fig. 6, *K* and *L*). Finally, we found that RSV also induced KIRA8-susceptible XBP1-Pol II binding to *GFPT2* gene at its intragenic superenhancer and to *MMP9* gene at its promoter (Fig. 6, *O* and *P*; Supplemental Fig. S7). Collectively, these data indicate that XBP1s can act as a direct regulator of the core EMT and HBP regulatory genes and cytokine and EMT/ECM-related genes, an interaction seemingly required for inducible RNA polymerase II binding and transcription.

### XBP1 Autoregulation May Contribute to Sustained Activation of the IRE1α-XBP1s Pathway

**Figure 7.**
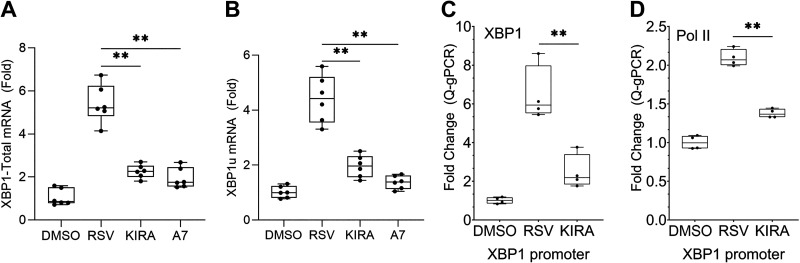
Autoregulation of *XBP1*. hSAECs were RSV infected (MOI = 1.0) for 24 h in the presence of DMSO, KIRA8 (KIRA), or ceapin-A7 (A7) at 10 μM. *A* and *B*: Q-RT-PCR of total XBP1 (*XBP1*-Total) and unspliced XBP1 (*XBP1u*). Shown is fold change of mRNA relative to mock infection (DMSO) in *n* = 3 independent experiments. Recruitment of XBP1 (*C*) and RNA Pol II (*D*) to *XBP1* promoter. XChIP was conducted using XBP1 or RNA Pol II-specific antibodies (XBP1, Cat. No. ab37152 at Abcam; RNA Pol II, Cat. No. ab26721 at Abcam). The XChIP-enriched genomic DNA was analyzed by Q-gPCR using *XBP1* promoter-specific primers (Table 2). Data are calculated as fold change relative to uninfected hSAECs (DMSO) and plotted as means ± ranges plus all data points of duplicate independent immunoprecipitates. Note the RNA Pol II dependence on IRE1α-XBP1 signaling. **P < 0.01, ANOVA. hSAECs, human small airway epithelial cells; MOI, multiplicity of infection; Q-RT-PCR, quantitative reverse transcription polymerase chain reaction; RSV, respiratory syncytial virus; XBP1, X-box binding protein 1.

Continuous synthesis of the precursor *XBP1u* mRNA is necessary for sustaining the production of XBP1s in response to UPR activation in RSV-infected cells. We observed that RSV infection significantly induces total XBP1 (Fig. 2*C*) and XBP1u (Supplemental Fig. S3) mRNA expression and that knock-down of IRE1α led to downregulation of XBP1 mRNA while downregulating XBP1s (Fig. 5, *A* and *C*), prompting us to test whether this induction is linked to activation of XBP1s. Strikingly, RSV-induced total XBP1 or XBP1u mRNA was markedly attenuated by inhibition of IRE1α-XBP1s or ATF6 pathway by small molecule inhibitors (Fig. 7, *A* and *B*). To further investigate whether XBP1s may autoregulate synthesis of its precursor, XChIP assays were used to examine whether RSV induces XBP1 binding to its own promoter. Remarkably, RSV induced a 6.9 ± 1.4-fold (*P* = 1.8E-04) increase in XBP1 binding to its own (*XBP1*) promoter, associated with a significant increase in RNA Pol II binding. This induction of RNA Pol II binding was XBP1-dependent because its abundance was downregulated by the reduction of XBP1s formation by KIRA8 (Fig. 2, *E* and *F* and Fig. 7, *C* and *D*). These results suggest that the IRE1α-XBP1s pathway is in a positive amplification loop to sustain the UPR in response to RSV infection.

### Sendai Virus Infection Activates the UPR and EMT

**Figure 8.**
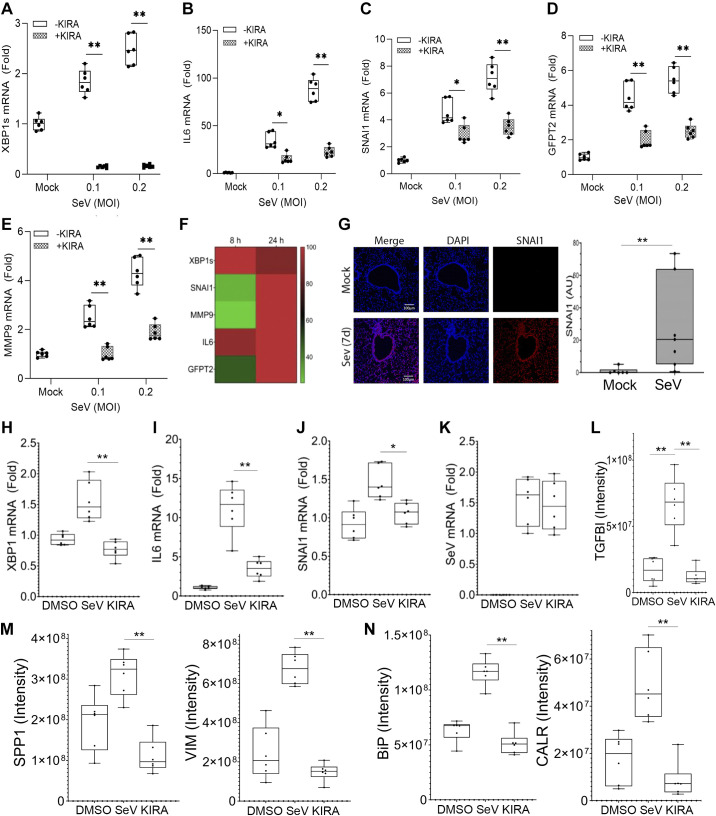
Sendai virus activates the UPR-HBP/EMT pathways in vitro and in vivo. *A–E*: hSAECs were infected with SeV at different MOI as indicated in the presence or absence of 10 µM KIRA8 (KIRA) for 24 h and Q-RT-PCR was performed. Shown is fold change of mRNA relative to mock infection for *XBP1s, IL6, SNAI1, GFPT2,* and *MMP9*. *P < 0.05, **P < 0.01, ANOVA with post hoc pairwise comparison. *F*: hierarchical clustering of *XBP1s*, *IL6*, and EMT signature genes. hSAECs were infected by SeV at 0.2 MOI for 8 or 24 h and fold change of mRNA relative to mock infection was determined by Q-RT-PCR. The temporal expression patterns of signature epithelial or mesenchymal genes in the cells were *z*-score-normalized and subjected to hierarchical clustering using Euclidian distance. Note the rapid induction of *XBP1s*/*IL6* and delayed increase in *GFPT2*, *SNAI1,* and *MMP9* (cf. Fig. 3, *G*–*I*). *G:* C57BL/6J black mice (both sexes) were infected with SeV for 7 days. IHC for SNAI1 in airways. Scale bar is 100 µM. *Right*: quantitation in multiple independent fields from different mice. ** *P* < 0.01, paired *t* test. *H–N*: C57BL/J6 black mice (both sexes) were administered SeV or vehicle (PBS) via the intranasal route. Randomly selected mice were then treated every day with the IRE1α inhibitor KIRA8 at 50 mg/kg/day (KIRA) for 3 days. Lung total RNA was harvested and used to measure *XBP1s* (*H*), *IL6* (*I*), *SNAI1* (*J*), and SeV (*K*) transcription by Q-RT-PCR. Fold change of mRNA relative to mock infection with means ± ranges plus all data points from individual mice are shown. *L–N*: LC-MS/MS analysis of BALF. Bars represent means ± ranges plus all data points of multiple mice. *P < 0.05, **P < 0.01, ANOVA with post hoc comparison. EMT, epithelial mesenchymal transition; HBP, hexosamine biosynthetic pathway; hSAECs, human small airway epithelial cells; IRE1α, inositol-requiring enzyme 1 α; MOI, multiplicity of infection; Q-RT-PCR, quantitative reverse transcription polymerase chain reaction; SeV, Sendai virus; SNAI1, Snail family transcriptional repressor 1; UPR, unfolded protein response.

Murine *respirovirus*, also known as Sendai virus (SeV), is a negative sense, single-stranded RNA virus of the family Paramyxoviridae. SeV infection in mice partially mimics the pathogenesis observed in human RSV LRTI; for example, primary viral replication occurs in the upper nasopharynx, followed by transmission into the trachea into the lungs, producing pneumonia. Like RSV, SeV replication causes inflammation, giant cell formation, and necrosis of the respiratory epithelium (6). In hSAECs, we observed that SeV infection triggers the activation of the IRE1α-XBP1s pathway, associated with the upregulation of *IL6*, *SNAI1*, *GFPT2*, and *MMP9* expression in an MOI-dependent manner, and sensitive to KIRA8 (Fig. 8, *A*–*E*). Of note, *XBP1* splicing and *IL6* expression were also upregulated as an early event followed by later expression of *SNAI1, GFPT2,* and *MMP9*, as shown in the heat map of mRNA expression in Fig. 8*F*, in a similar temporal relationship as observed in RSV infection (cf. Fig. 3, *G*–*I*).

To determine whether SeV infection induces mucosal EMT, C57BL/J6 mice (*n* = 5 animals/group, both sexes) were infected with SeV (10^4^ PFU, Cantell Strain) and harvested lungs for IF and RT-PCR after 7 days. In this model, SeV replication occurs from 3 to 5 days and is cleared. We observed a variable, but persistent and significant upregulation of SNAI1 expression in the airway epithelium 7 days after SeV infection (median of 20-fold induction; Fig. 8*G* with quantification). Further, we infected C57BL/6 mice with SeV in the absence or presence of KIRA8 (50 mg/kg QOD) for 3 days to assess the effect of inhibiting the IRE1α-XBP1s pathway to the cellular responses to SeV infection. As shown by Q-RT-PCR with lung total RNA, KIRA8 administration blocked SeV-induced upregulation of XBP1s (Fig. 8*H*), IL6 (Fig. 8*I*), and SNAI1 (Fig. 8*J*) mRNAs without affecting SeV transcription (Fig. 8*K*).

Proteomics analysis of the bronchoalveolar lavage fluid (BALF) was also conducted by LC-MS/MS. We found that markers of EMT, including TGFβ-induced protein ig-h3 (TGFBI, Fig. 8*L*), osteopontin/SPP1, and VIM (Fig. 8*M*) were upregulated in the SeV-infected mice and inhibited by the administration of KIRA8. Similarly, markers of the UPR, such as HSP5A/Bip and CALR were significantly elevated whose accumulation was prevented by KIRA8 administration (Fig. 8*N*). We also noted abundance of other ECM remodeling proteins (LGALS3BP and others, not shown) was also increased in the BALF collected from SeV-infected mice and reduced by KIRA8. These data indicate that paramyxovirus infection induces IRE1α-XBP1 arm of the UPR, which mediates inflammatory response, HBP, the EMT program, and the release of ECM proteins in the mucosa in vivo.

## DISCUSSION

RSV is a major human pathogen responsible for substantial acute morbidity and hospitalizations worldwide. LRTIs are associated with long-term decreased pulmonary function and obstructive patterns of lung function through largely unknown mechanisms (18, 19). The mechanisms of how paramyxovirus infections are associated with long-term structural remodeling are underexplored and not fully understood. Building on earlier studies that innate inflammation mediates changes in glycolysis and accumulation of UDP metabolites, we explore the relationship between viral replication, HBP, the UPR, EMT and ECM remodeling in a disease-relevant model of lower airway epithelial cell. Small airway epithelial cells have gained attention because of their role in the development of obstructive lung disease (47), asthma (48), interstitial fibrosis (49), and may play an important role in remodeling in response to viral LRTI. In fatal cases of LRTI, RSV replication is found in small bronchiolar epithelium (4). That this cellular phenotype plays a functional role in RSV LRTI was confirmed by tissue-selective genetic knockout of innate signaling in SAECs which blocks neutrophilia, airway obstruction, and disease manifestations (30). Moreover, systems-level findings have shown that SAECs produce Th2-polarizing, mucogenic, and profibrotic cytokines that mediate the pathogenesis of LRTI (20) and undergo cell state transitions downstream of the innate pathway (39, 50). Here, we extend our understanding of the antiviral response of SAECs to a virus-induced glucose metabolic reprogramming and EMT program. Using submerged cell cultures, we demonstrate here that both primary and immortalized human small airway epithelial cells activate the IRE1α-XBP1 arm of UPR in response to RSV infection, which induces the gene expression of HBP rate-limiting enzymes and EMT core transcription regulators. We found that viral-induced XBP1s binds and recruits RNA polymerase II to the promoters or enhancers of IL6, SNAI1, GFPT2, and MMP9 genes, supporting the new mechanism that RSV-induced XBP1-UPR reprograms glucose metabolism, sustains the EMT process, and triggers ECM remodeling. Although these findings will need to be replicated in organoid cultures and models of RSV infection, we believe they have important implications in the interface between paramyxovirus infections and airway remodeling.

### The HBP Pathway Plays Dual Roles in Viral Infection

The HBP is a homeostatic response to EMT that upregulates the cellular capacity for *N*-glycosylation of ECM proteins essential in remodeling the basal lamina. Mechanistically, we provide evidence that replicating paramyxovirus infection induces longer term mucosal HBP, EMT persistence, and evidence of ECM remodeling after viral clearance. Previous work has shown that RSV subverts core cellular metabolic pathways to support ribonucleotide production (51). In this study, we observe RSV perturbs glycolysis via the HBP in hSAECs, enhancing UDP-GlcNAc accumulation and protein *N*-glycosylation in an IRE1α-dependent manner. We focus on expression of GFPT1 and GFPT2 because these enzymes are rate-limiting controlling flux of glucose into the hexosamine pathway and are responsible for the synthesis of UDP-GlcNAc, a sugar donor for *N*-glycosylation. *N*-glycosylation is important for proteostasis, promoting the folding of ECM proteins and relief of ER stress. In addition, activation of the HBP may be highly advantageous for the virus by enhancing the processing of RSV F and G glycoproteins, the key structural components of the mature virion, and the SH glycoprotein, important in virion assembly (52).

Earlier studies found that inhibition of IRE1α resulted in enhanced F and G glycoprotein production, suggesting that IRE1α may play a direct antiviral role (53). We are not able to confirm this finding since KIRA8 has no significant effect on RSV replication in hSAEC or SeV in vivo, and silencing IRE1α mRNA does not affect RSV transcription or infectivity.

### Type 2 EMT in the Airway Is under Coordinate Control of Virus-Inducible Signaling Pathways

The airway epithelium rapidly reprograms its genome in response to environmental signals via innate signaling. Recent work has implicated a central role of the innate immune response effector, NF-κB, in coupling viral inflammation and cellular reprogramming (39, 54, 55). RNA sequencing studies have shown that NF-κB is a master transcription factor of EMT and directly controls *SNAI1* expression (38, 39). In differentiated cells, expression of SNAI-ZEB module is regulated by double-negative feedback loop with the miR-53 and miR-200 microRNAs (39). Activation of SNAI1 disrupts this negative feedback loop, resulting in transition into EMT. Our findings reveal that IRE1α-XBP1 is one of the pathways controlling EMT through regulating the expression of SNAI1 and shed further light into how ER stress is coupled to EMT.

Using a highly sensitive two-step chromatin immunoprecipitation assay, we observe that XBP1 interacts with the *SNAI1, IL6,* and *GFPT2* promoters or enhancer in RSV-infected cells. Interestingly, these genes do not have a classic unfolded protein response element, suggesting that XBP1s binding may be indirectly mediated by other inducible sequence-specific binding factors, consistent with the findings of others (45). In addition to XBP1s directly interacting with the GFPT2, SNAI1, and FN1 promoters, additional mechanisms may contribute to the IRE1α-dependent expression. IRE1α is known to activate NF-κB and AP-1 transcription factors through its kinase domain (56, 57), both of which are widely involved in gene regulation in inflammation, EMT, and ECM remodeling (39, 58, 59). Second, the IRE1α-XBP1s pathway plays an essential role in maintaining the cellular secretory apparatus in addition to facilitating protein folding in the ER (60). Enhancement in both the secretory pathway and ER capacity may facilitate cytokine secretion that indirectly activating cytokine-transcription factor positive loops (61). A recent phosphoproteomics study by us further illustrated complex cross talk between the IκB kinase and XPB1s, where we discovered that IKKβ forms a complex with and phosphorylates XBP1s in TGFβ-induced HBP (62). The contributions of cross talk pathways to the direct actions of XBP1 in paramyxovirus infection will require further investigation.

### Autoregulation of XBP1 in Paramyxovirus-Induced UPR

Our data indicate that spliced XBP1 binds to- and transactivates the XBP1 promoter by recruiting RNA Pol II. We interpret this interesting finding, first to our knowledge, that the UPR employs positive autoregulation to provide sufficient amount of XBP1 mRNA for sustaining the UPR and HBP while ER stress persists. Concurrently, RSV replication also causes substantial induction of IRE1α protein through unidentified mechanisms (Fig. 2*J*). Enhanced, coordinate expression of the IRE1α and XBP1 precursors may play a significant role in sustaining robust RSV-mediated UPR signaling.

### Activation of the UPR in RSV Infection

We observed that both RSV and SeV induce glycoprotein retention in the ER and rapidly activate the UPR in vitro and in vivo. Although our study was not designed to address the question how RSV activates the UPR, we suspect that the proximal signals activating ER stress in RSV infection may be from the burst of newly synthesized virally encoded glycoproteins in the ER or through disruption of ER membranes by viral replication. Previous work showed that RSV F directly complexes with binding immunoglobulin protein (BiP) (63), providing a mechanism for direct activation of the UPR pathways. This study has not been performed with murine respirovirus F to our knowledge. In addition, in response to RSV infection, epithelial expression and secretion of inflammatory and antiviral cytokines are one of the most robust and rapid innate immune responses that could contribute to early ER stress and UPR activation.

Our data suggest that paramyxovirus replication activates the evolutionary conserved IRE1α-XBP1 pathway, processing of ATF6 and PERK activation (53). Our work extends these studies linking the highly conserved arm of the UPR, IRE1α-XBP1s pathway to the HBP and EMT. Although we observe the hallmark of PERK activation by eIF2α phosphorylation, responsible for translational shut-off (64), it is notable that global protein synthesis is unaffected in RSV-infected cells (43). More work will be required to understand how cells escape translational shut-off despite eIF2α phosphorylation.

### Viral Induction of UPR, EMT, and ECM Remodeling

We believe our elucidation of the UPR activation provides a mechanism by which severe paramyxovirus infection promotes cell state transition and ECM remodeling. Our earlier studies implicated MMP9 as a major trigger of RSV-induced ECM remodeling and myofibroblast expansion (37). Our findings here extend this understanding to show that RSV induces coordinate production of ECM products and remodeling enzymes in vitro including COL1, FN1, and MMP9 (Fig. 3*F*) mediated by the IRE1α-XBP1 pathway (Fig. 4*D*). Moreover, EMT and active ECM remodeling is seen in response to SeV infection, with the detection of TGFBI, SPP1, VIM, CALR in the BALF fluids, providing unequivocal evidence of cell state transition and active ECM remodeling. Because this study was focused on the early molecular triggers of airway remodeling, longer studies will be required to understand the extent of structural remodeling that may occur after paramyxovirus infection.

It is well established that viral URIs are responsible for the majority of acute exacerbations of wheezing and asthma, and that these acute exacerbations may lead to changes in pulmonary function (65, 66). We speculate that activation of the UPR with EMT and ECM remodeling may be an early molecular signature of viral-induced remodeling.

In summary, these data indicate that paramyxovirus-induced UPR is central to metabolic adaptation, cell state transition, and ECM remodeling in paramyxovirus infection. This may have implications for virus-induced structural airway remodeling.

## SUPPLEMENTAL DATA

Supplemental Figs. S1–S7: https://doi.org/10.6084/m9.figshare.14912631.v1.

## GRANTS

This work was partially supported by the National Institutes of Health Grants AI062885 (to R.P.G. and A.R.B.), R21AI133454 (to Y.Z. and A.R.B.), and NCATS UL1TR002373 (to A.R.B.).

## DISCLAIMERS

The funders had no role in the design of the study; in the collection, analyses, or interpretation of data; in the writing of the manuscript; or in the decision to publish the results.

## DISCLOSURES

No conflicts of interest, financial or otherwise, are declared by the authors.

## AUTHOR CONTRIBUTIONS

D.Q. and A.R.B. conceived and designed research; D.Q., M.S., X.X., Y.Z., and A.R.B. performed experiments; D.Q., M.S., X.X., R.P.G., and Y.Z. analyzed data; D.Q., Y.Z., and A.R.B. interpreted results of experiments; D.Q., X.X., Y.Z., and A.R.B. prepared figures; D.Q. and A.R.B. drafted manuscript; D.Q., M.S., Y.Z., and A.R.B. edited and revised manuscript; D.Q., M.S., X.X., Y.Z., and A.R.B. approved final version of manuscript.
